# Inhibition of HDAC4 by GSK3β leads to downregulation of KLF5 and ASK1 and prevents the progression of intravertebral disc degeneration

**DOI:** 10.1186/s13148-021-01005-9

**Published:** 2021-03-10

**Authors:** Lin Xiao, Dongping Gong, Loufeng Liang, Anwei Liang, Huaxin Liang, Xiayi Xu, Hongli Teng

**Affiliations:** Department of Pain, Guangxi University of Chinese Medicine, Guangxi International Zhuang Medicine Hospital, No. 8, Qiuyue Road, Wuxiang New District, Nanning, 530000 Guangxi Zhuang Autonomous Region People’s Republic of China

**Keywords:** GSK3β, KLF5, ASK1, HDAC4, Intravertebral disc degeneration

## Abstract

**Background:**

Intervertebral disc degeneration (IDD) is a major cause of lower back pain. This study aimed at exploring the effects of histone deacetylase 4 (HDAC4) and its upstream and downstream signaling molecules on IDD development.

**Methods:**

A murine IDD model was established by inducing a needle puncture injury to the vertebrate, whereupon we isolated and transfected of nucleus pulposus (NP) cells. Disc height index (DHI) of the mice was determined by X-ray tomography, while the pain experienced by the IDD mice was evaluated by mechanical and thermal sensitivity tests. Next, the interaction between GSK3β and HDAC4 as well as that between HDAC4 and KLF5 acetylation was assessed by co-immunoprecipitation, while the promoter region binding was assessed identified by chromatin immunoprecipitation. By staining methods with TUNEL, Safranin O fast green, and hematoxylin and eosin, the NP cell apoptosis, degradation of extracellular matrix, and morphology of intervertebral disc tissues were measured. Furthermore, mRNA and protein expressions of GSK3β, HDAC4, KLF5, and ASK1, as well as the extent of HDAC4 phosphorylation, were determined by RT-qPCR and Western blotting.

**Results:**

GSK3β was identified to be downregulated in the intervertebral disc tissues obtained from IDD mice, while HDAC4, KLF5, and ASK1 were upregulated. HDAC4 silencing alleviated IDD symptoms. It was also found that GSK3β promoted the phosphorylation of HDAC4 to increase its degradation, while HDAC4 promoted ASK1 expression through upregulating the expression of KLF5. In IDD mice, GSK3β overexpression resulted in increased DHI, inhibition of NP cell apoptosis, alleviation of disc degeneration, and promoted mechanical and thermal pain thresholds. However, HDAC4 overexpression reversed these effects by promoting ASK1 expression.

**Conclusion:**

Based on the key findings of the current study, we conclude that GSK3β can promote degradation of HDAC4, which lead to an overall downregulation of the downstream KLF5/ASK1 axis, thereby alleviating the development of IDD.

**Supplementary Information:**

The online version contains supplementary material available at 10.1186/s13148-021-01005-9.

## Background

Intervertebral disc degeneration (IDD) is one of the most common causes of lower back pain and related disability [1, 2]. Back pain is a common chronic medical problem worldwide, such that over 80% of adults are estimated to have suffered from back pain at some point in their lives [3]. In adults below the age of 45 years, back pain has been identified as the most common cause of limited activity [3]. The causes of back pain include vertebral traumatic injury or chronic persistent stress. Chronic back pain is associated with increased economic and social burden and is one of the most common causes of absence from work [4]. Intervertebral discs consist of two major components, namely the annulus fibrosus and nucleus pulposus (NP). The NP is centrally located in the intervertebral disc and mainly consists of proteoglycans, collagen II, and elastic fibers [2]. The molecular mechanisms of IDD are yet to be fully understood, but are believed to involve increased degradative enzymes, proinflammatory cytokines, and loss of matrix proteins [5]. Therefore, the present study was conducted aiming to determine the molecular mechanism that contributes to IDD in NP tissues.

Histone deacetylase 4 (HDAC4) is regarded as an important mediator of disease processes due to its essential function on the transcriptional regulation and cell cycle progression [6]. In addition, it has also been reported that histone acetylation/deacetylation affects chromosome structure and access of transcription factors to DNA [7]. A recent study revealed novel findings suggesting the involvement of HDAC4 in end plate chondrocyte degeneration [8]. That study showed that HDAC4 inhibition resulted in the alleviation of end plate chondrocyte degeneration, highlighting that HDAC4 may promote IDD [8]. In the current study, we aimed to further determine whether and how HDAC4 promotes IDD.

Interestingly, as previously reported, glycogen synthase kinase-3β (GSK3β) could promote HDAC4 degradation by phosphorylating HDAC4 [9], while it has also been illustrated that GSK3β inhibited IDD [10]. Therefore, the potential upstream inhibitory role of GSK3β in IDD progression through the inhibition of HDAC4 was evaluated. In addition, the downstream signaling pathway of HDAC-mediated IDD was determined. A previous study showed that HDAC4 promoted transcriptional function by removing the acetylation of Kruppel-like factor 5 (KLF5) protein through its deacetylase activity [11]. As a transcription factor, KLF5 promotes the expression of apoptosis signal-regulating kinase 1 (ASK1, also known as MAP3K5) [12], while ASK1 has been shown to promote NP cell apoptosis and promote neuropathic pain [13, 14]. Thus, on the basis of the aforementioned previous findings, we presumed the possible involvement of KLF5/ASK1 axis in the effect of GSK3β-regulated HDAC4 on IDD, which is the main issue needed to be conferred in this research.

## Results

### Silencing of HDAC4 prevents IDD

**Fig. 1:**
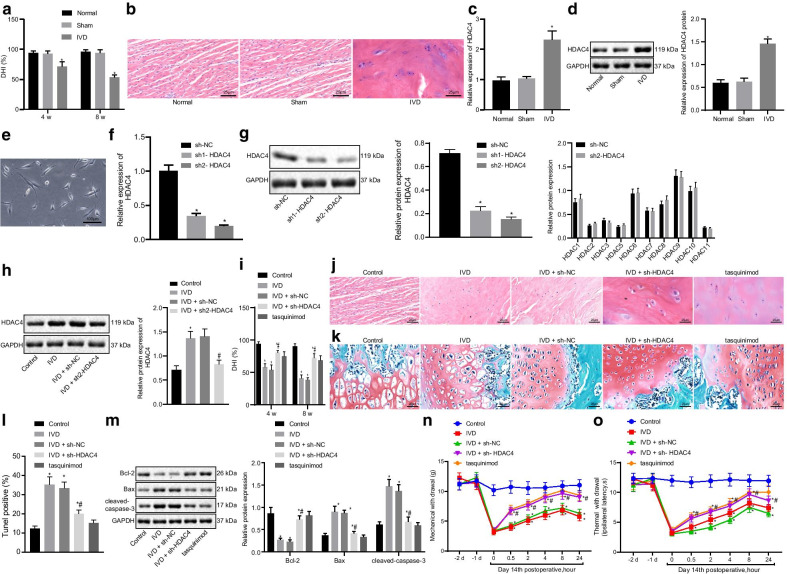
Silencing of HDAC4 relieves IDD. **a** Disc height index (DHI). **b** Representative micrographs showing disc tissues after H&E staining (400×). **c** HDAC4 mRNA expression determined by RT-qPCR. *p < 0.05 versus control. **d** HDAC4 protein expression determined by Western blotting; *p < 0.05 versus control. **e** Identification of NP cells. **f** Silencing efficiency of HDAC4 expression (mRNA) determined by RT-qPCR; *p < 0.05 versus NC. **g** Silencing efficiency of HDAC4 expression (protein) determined by Western blotting; *p < 0.05 versus NC. **h** The in vivo silencing efficiency of sh2-HDAC4 determined by Western blotting; **i** Disc height index (DHI). **j** Representative micrographs showing disc tissue after H&E staining (400×). **k** Representative micrographs showing disc tissue after Safranin O fast green staining (400×). **l** Apoptosis determined by TUNEL assay; **m** Bcl-2, Bax, and cleaved caspase-3 protein expression determined by Western blotting. **n** Mechanical pain threshold. **o** Thermal sensitivity threshold. *p < 0.05 versus control; #*p* < 0.05 versus sh-NC. *n* = 9 for animal experiments; cell experiments were performed in triplicate

As previously reported, HDAC4 promotes the development of IDD [15]. With the attempt to further explore the effect of HDAC4 on the IDD and its mechanism, IDD mouse models were established. Then, to verify the success of IDD mouse model, we measured the disc height index (DHI) in IDD mice on weeks 4 and 8 following the operation, the results of which showed an increase in DHI (Fig. 1a). In addition, in the control and sham-operated mice, the disc structure was normal, chondrocytes were abundant, fibrous ring was intact, and boundary between the fibrous ring as well as the NP was clear (Fig. 1b). However, intervertebral disc tissue structure was abnormal, discs were fused or deformed, and chondrocytes formed typical cell cluster degeneration in IDD mice (Fig. 1b). These results showed that IDD model was successfully established.

After that, the expression of HDAC4 in the mouse model was detected by reverse transcription quantitative polymerase chain reaction (RT-qPCR) and Western blotting, which revealed that HDAC4 mRNA and protein expressions were increased in NP tissues of IDD mice when compared to control or sham mice, while there was no difference in the mRNA and protein expressions of HDAC4 between control and sham mice (Fig. 1c, d). NP cells were spindle-shaped or horn-shaped, with long cytoplasmic protrusions (Fig. 1e). Two short hairpin RNA (sh)-HDAC4 sequences (sh1-HDAC41 and sh2-HDAC4) were then used to knock down the HDAC4 expression in NP cells. It was observed that both sh1-HDAC41 and sh2-HDAC4 significantly reduced the transcription level (Fig. 1f) and protein level (Fig. 1g) of HDAC4, while sh2-HDAC4 was more effective and therefore selected for further experiment. Then, as the in vivo experiments revealed, sh2-HDAC4 could successfully produce effects in vivo (Fig. 1h). In addition, HDAC4 silencing was identified to increase DHI in IDD mice (Fig. 1i), which also improved the morphology of intravertebral discs in IDD mice (Fig. 1j). Intervertebral disc had reduced deformities, abnormal chondrocytes, and degenerative cells. In response to the positive control tasquinimod, the abnormal tissue structure of intervertebral disc was rescued, chondrocytes were abundant, and the boundary between annulus fibrosus and NP was clear. Safranin O fast green positive tissue was present (Fig. 1k). Mucin, chondrocytes, and mast cell granules were stained with an orangish red color. NP, fibrous rings, and cartilage plates all stained red, indicating proteoglycan was abundant. Red-stained structures were reduced in IDD mice, suggesting decreased proteoglycan content. HDAC4 silencing improved disc structure and increased red-stained cells.

Moreover, apoptosis in NP cells was found to be significantly increased in IDD mice that were normalized by HDAC4 silencing (Fig. 1l). Moreover, the expression of B-cell lymphoma 2 (Bcl-2) was significantly decreased, while Bcl-2-associated X protein (Bax) and cleaved-caspase-3 expression was increased in NP tissues from IDD mice (Fig. 1m). HDAC4 silencing normalized the effect of IDD. Furthermore, mechanical pain (Fig. 1n) and thermal hyperalgesia thresholds (Fig. 1o) were significantly reduced at 2, 4, and 8 h when compared with control mice. HDAC4 silencing significantly improved these pain thresholds. The above results showed that silencing of HDAC4 alleviated IDD.

### HDAC4 promotes ASK1 expression through KLF5

**Fig. 2:**
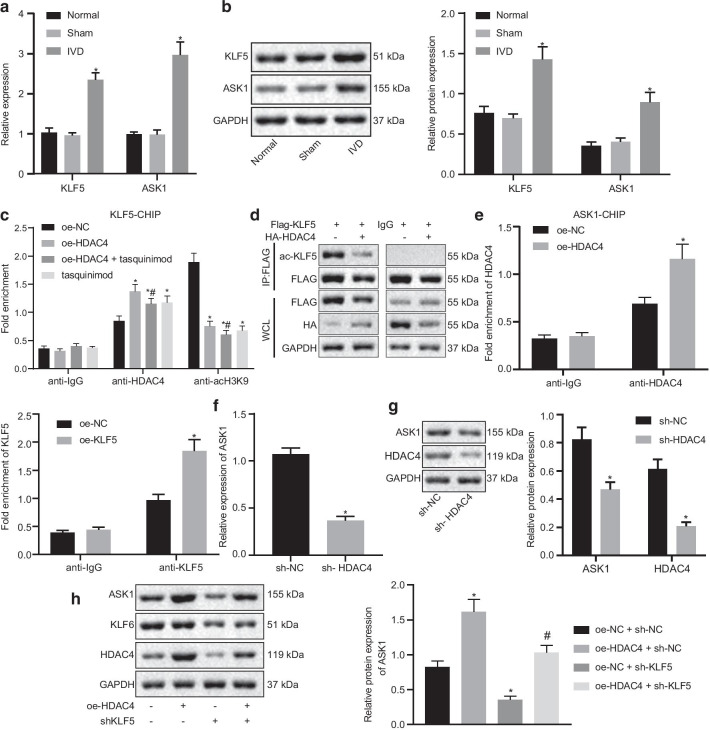
HDAC4 promotes ASK1 expression through KLF5. **a** KLF5 and ASK1 mRNA expression. *p < 0.05 versus control. **b** KLF5 and ASK1 protein expression determined by Western blotting; *p < 0.05 versus control. **c** Binding between HDAC4 and the KLF5 promoter determined by ChIP; KLF5-ChIP is the promoter chip. *p < 0.05 versus oe-NC. **d** KLF5 acetylation determined by IP. **e** Binding of gene promoter determined by ChIP; ASK1-ChIP is the promoter chip. *p < 0.05 versus oe-NC. **f** ASK1 mRNA expression determined by RT-qPCR. **g** ASK1 protein expression determined by Western blotting; *p < 0.05 versus sh-NC. **h** ASK1 protein expression determined by Western blotting following oe-HDAC4, sh-KLF5 or both. *Indicates *p* < 0.05 and **indicates *p* < 0.01. *n* = 9 for animal experiments; cell experiments were performed in triplicate

A previous study revealed that HDAC4 can remove the acetylation of KLF5 protein through its deacetylase function and promote its transcription function [16]. At the same time, KLF5 transactivation can promote ASK1 expression [12]. Thus, we further examined the potential downstream signaling molecules of HDAC4 (KLF5 and ASK1) in IDD. The mRNA and protein expressions of KLF5 and ASK1 were increased in NP tissues of IDD mice when compared to control mice (Fig. 2a, b). In addition, chromatin immunoprecipitation (ChIP)-PCR experimental results showed that HDAC4 overexpression significantly enriched HDAC4 in the KLF5 promoter region (Fig. 2c). Moreover, IP showed that HDAC4 overexpression resulted in the significant inhibition of the acetylation of KLF5 (Fig. 2d). ChIP experiments further showed that HDAC4 interacted with ASK1. KLF5 transcription factor bound to the promoter region of ASK1 (Fig. 2e). In contrast, the results of reverse transcription quantitative polymerase chain reaction (RT-qPCR) (Fig. 2f) and Western blotting (Fig. 2g) revealed that HDAC4 silencing reduced the mRNA and protein expressions of ASK1, whereas protein expression of ASK1 was reduced following HDAC4 silencing (Fig. 2g). Besides, overexpression of HDAC4 increased ASK1 protein expression, while knockdown of KLF5 reduced the ASK1 expression. Dual treatment with overexpression (oe)-HDAC4 and sh-KLF5 decreased ASK1 protein expression compared with oe-HDAC4 alone and increased the protein expression compared with sh-KLF5 alone (Fig. 2h). The above results suggested that HDAC4 promoted ASK1 expression through KLF5 in IDD mice.

### HDAC4 promotes ASK1 expression aggravates IDD

**Fig. 3:**
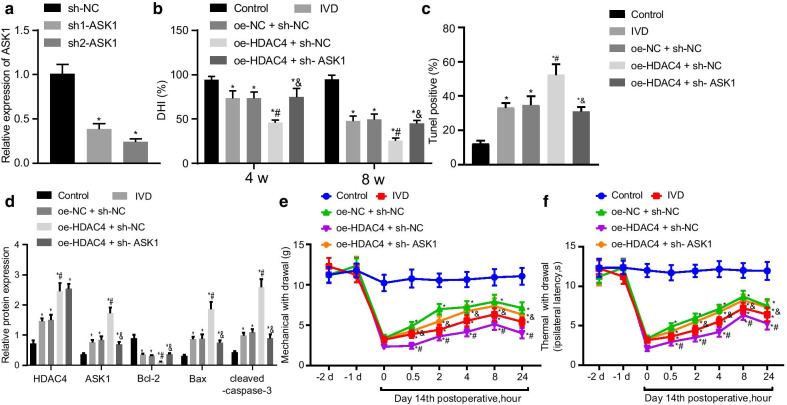
HDAC4 elevates ASK1 expression and thus promotes IDD. **a** ASK1 silencing efficiency determined by RT-qPCR; *p < 0.05 versus sh-NC. **b** Changes in disc height index (DHI). **c** Apoptosis reflected by TUNEL-positive cells. **d** HDAC4, ASK1, Bcl-2, Bax, and cleaved caspase-3 protein expression determined by Western blotting. **e** Mechanical pain threshold. **f** Thermal sensitivity threshold; *p < 0.05 versus control; #*p* < 0.05 versus oe-NC + sh-NC; &*p* < 0.05 versus oe-HDAC4 + sh-NC. *n* = 9 for animal experiments; cell experiments were performed in triplicate

After establishing the promoting effect of HDAC4 on ASK1 expression, we further investigated the effect of HDAC4 on IDD by regulating ASK1. First, we detected the silencing efficiency of ASK1 expression using RT-qPCR, which showed that both ASK1 silencing sequences significantly inhibited the expression of ASK1 (Fig. 3a). sh2-ASK1 was more effective and was therefore selected for subsequent experiments. HDAC4 overexpression decreased DHI in IDD mice, which was normalized following ASK silencing (Fig. 3b). Hematoxylin and eosin (H&E) staining showed that HDAC4 overexpression caused intervertebral disc tissue structure to be disordered, with fused or deformed intervertebral discs fused and abnormality of intervertebral disc chondrocytes, forming a typical cell cluster degeneration (Additional file 1: Figure S1A). Safranin O fast green-positive tissue was present upon HDAC4 overexpression (Additional file 2: Figure S2A), showing atrophy in NP tissue, narrowed intervertebral discs, and less red staining overall than in control, indicating reduced proteoglycan content. ASK silencing normalized intervertebral disc NP morphology and increased red staining. HDAC4 overexpression increased apoptosis in IDD mice that was normalized by ASK1 silencing (Fig. 3c). Western blotting revealed that HDAC4 overexpression decreased Bcl-2 protein expression, while increasing ASK1, Bax, and cleaved caspase-3 protein expression (Fig. 3d). These changes were normalized as a result of ASK1 silencing, with the exception of HDAC4 expression, which was not susceptible to this effect. Moreover, HDAC overexpression significantly reduced mechanical pain (Fig. 3e) and thermal hypersensitivity (Fig. 3f) thresholds at 2, 4, and 8 h. ASK1 silencing alleviated reduced pain thresholds. These results indicated HDAC4 increased ASK1 expression and promoted IDD.

### GSK3β promotes HDAC4 phosphorylation and degradation

**Fig. 4:**
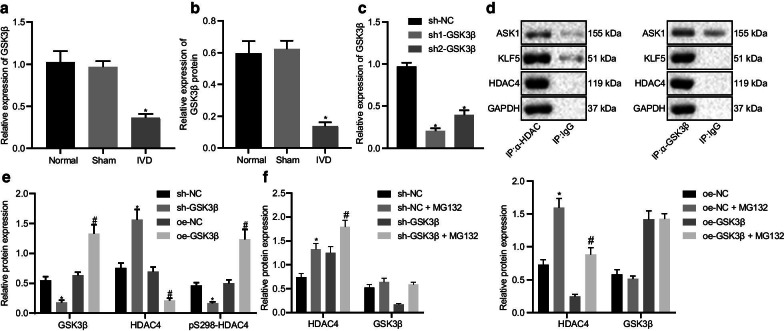
GSK3β promotes HDAC4 phosphorylation and degradation. **a** GSK3β mRNA expression. **b** GSK3β protein expression determined by Western blotting; *p < 0.05 versus control. **c** GSK3β silencing efficiency determined by RT-qPCR; *p < 0.05 versus sh-NC. **d** Interaction between GSK3β and HDAC4 determined by Co-IP. **e** HDAC4 phosphorylation level determined by Western blotting after GSK3β knockdown or overexpression. **f** HDAC4 and GSK3β protein expression determined by Western blotting after GSK3β knockdown, overexpression, and/or proteasome inhibitor MG132 treatment; *p < 0.05 versus sh-NC; #*p* < 0.05 versus oe-NC. Cell experiments were performed in triplicate

GSK3β is capable of promoting HDAC4 degradation through its phosphorylation [9]. Meanwhile, GSK3β can prevent the occurrence of IDD [10]. To verify the possible interplay between GSK3β and HDAC4 in IDD, a series of experiments were conducted. Results of RT-qPCR and Western blotting showed mRNA (Fig. 4a) and protein (Fig. 4b). GSK3β expression was significantly reduced in NP tissues of IDD mice. We then tested the silencing efficiency of GSK3β. Both GSK3β silencing sequences significantly inhibited the expression of GSK3β (Fig. 4c). sh1-GSK3β was selected for further experiment because it was more effective. Co-immunoprecipitation (Co-IP) experimental results showed that GSK3β interacted with HDAC4 (Fig. 4d). Moreover, results of Western blotting revealed that GSK3β silencing led to an increase in HDAC4 protein expression while decreased pS298-HDAC4 protein expression (Fig. 4e). GSK3β overexpression, in contrast, decreased HDAC4 expression while increased pS298-HDAC4 levels. After treatment with the proteasome inhibitor MG132, inhibition of GSK3β reduced the degradation of HDAC4, while GSK3β overexpression promoted the degradation of HDAC4 (Fig. 4f). These results showed that GSK3β was downregulated in IDD mice and GSK3β promoted HDAC4 degradation.

### GSK3β alleviates IDD by inhibiting HDAC4 expression

**Fig. 5:**
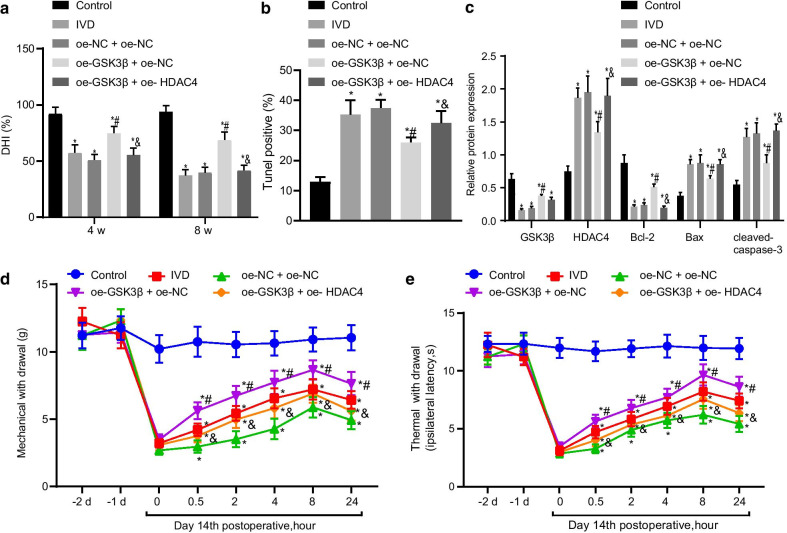
GSK3β alleviates IDD by inhibiting HDAC4 expression. **a** Changes in the disc height index (DHI). **b** Apoptosis determined by TUNEL assay (200×). **c** GSK3β, HDAC4, Bcl-2, Bax, and cleaved caspase-3 protein expression determined by Western blotting. **d** Mechanical pain threshold. **e** Thermal sensitivity threshold; *p < 0.05 versus control; #*p* < 0.05 versus oe-NC + oe-NC; &*p* < 0.05 versus oe-GSK3β + oe-NC. *n* = 9 for animal experiments; cell experiments were performed in triplicate

Then we proceeded to elucidate the mechanism by which GSK3β-mediated HDAC4 degradation affects IDD. GSK3β overexpression increased DHI in IDD mice, while further HDAC4 overexpression reversed the trend (Fig. 5a). Moreover, GSK3β overexpression improved the morphology of intervertebral disc tissues but further HDAC4 overexpression worsened the morphology (Additional file 1: Figure S1B). GSK3β overexpression caused increased red staining in intervertebral disc tissues in IDD mice after Safranin O fast green staining (Additional file 2: Figure S2A). However, HDAC4 overexpression worsened NP tissues and decreased red staining in IDD mice. GSK3β overexpression reduced apoptosis of NP cells in IDD mice that was reversed by HDAC4 overexpression (Fig. 5b). GSK3β overexpression decreased Bcl-2 while increased Bax and cleaved-caspase-3 protein expression in NP tissues (Fig. 5c). HDAC4 overexpression reversed these effects. GSK3β overexpression increased the threshold of mechanical pain (Fig. 5d) and hyperalgesia (Fig. 5e) at 2, 4, and 8 h. HDAC4 overexpression aggravated the pain of disc degeneration. These results indicated that GSK3β promoted the degradation of HDAC4 and thus alleviated the IDD, while its inhibition reduced the degradation of HDAC4, aggravating IDD.

### GSK3β alleviates IDD by inhibiting ASK1

**Fig. 6:**
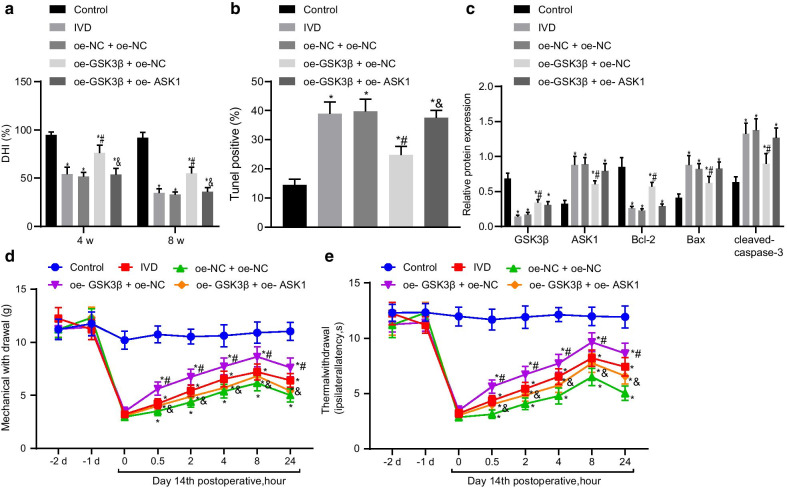
GSK3β alleviates IDD by inhibiting ASK1 expression. **a** Disc height index (DHI). **b** Cell apoptosis determined by TUNEL assay. **c** GSK3β, ASK1, Bcl-2, Bax, and cleaved caspase-3 protein expression determined by Western blotting. **d** Mechanical pain threshold. **e** Thermal sensitivity threshold; *p < 0.05 versus control; #*p* < 0.05 versus oe-NC + oe-NC; &*p* < 0.05 versus oe-GSK3β + oe-NC. *n* = 9 for animal experiments; cell experiments were performed in triplicate

Finally, we aimed to determine whether GSK3β can arrest the IDD through ASK1. GSK3β overexpression increased DHI in IDD mice significantly, which was inhibited secondary to ASK1 overexpression (Fig. 6a). H&E staining showed that GSK3β overexpression improved IDD by improving the morphology of NP tissues (Additional file 1: Figure S1C). However, the addition of ASK1 overexpression worsened the structure of NP tissues. Safranin O fast green staining showed an abundant red staining in IDD mice with overexpressed GSK3β (Additional file 2: Figure S2C). However, red staining was significantly reduced by ASK1 overexpression. GSK3β overexpression reduced apoptosis in NP tissues that was increased by ASK1 overexpression (Fig. 6b). Moreover, results of Western blotting revealed that GSK3β overexpression elevated Bcl-2 protein expression and reduced ASK1, Bax, and cleaved-caspase-3 protein expression (Fig. 6c), while the addition of ASK1 overexpression reversed these effects except had no effect on GSK3β expression. GSK3β overexpression significantly increased the thresholds of mechanical pain (Fig. 6d) and thermal (Fig. 6e) pain at 2, 4, and 8 h. ASK1 overexpression reduced these pain thresholds. These results showed that GSK3β alleviated IDD via ASK1 inhibition.

## Discussion

The present study revealed several key findings. Firstly, HDAC4 expression was increased in IDD. HDAC4 silencing alleviated IDD by increasing DHI, improving morphology of NP tissue, decreasing NP cell apoptosis, and reducing pain. Next, HDAC4 was found to increase the expression of KLF5, resulting in an increase in ASK1 expression. HDAC4 also promoted IDD, as indicated by the decrease in DHI, and increase in NP cell apoptosis and pain. These effects of HDAC4 were reversed secondary to ASK1 silencing. In addition, IDD presented with reduced GSK3β expression. GSK3β overexpression degraded HDAC4 and hence improved IDD; however, this effect was reversed by either HDAC4 overexpression or ASK1 overexpression. Collectively, these results suggest that GSK3β degraded HDAC4 to alleviate IDD through KLF5 and ASK1. Therefore, our findings showed that GSK3β could potentially serve as a novel therapeutic target for slowing the progression of IDD and back pain and this study paves the way for further investigations on the topic.

The thorough understanding of the mechanism underlying the occurrence of IDD can aid the development of a novel treatment for lower back pain, as IDD is a major cause of this condition [1, 3]. In this study, IDD mice were established by inducing vertebral traumatic injury (needle puncture) in mice. Based on our results, HDAC4 silencing increased DHI, improved the morphology of NP tissues, reduced NP cell apoptosis, and pain, suggesting that HDAC4 was involved in the progression of IDD. These results were in line with a recent study that showed that HDAC4 was involved in end plate chondrocyte degeneration [8]. With the exception of these two studies, no further findings have linked HDAC4 to IDD. Therefore, these results provide strong evidence that require further investigations. On the other hand, previous studies have shown that increased oxidative stress and inflammatory response might account for the progression of IDD [17].

Another important finding was that GSK3β degrades HDAC4 in IDD, suggesting GSK3β may be an upstream regulator of HDAC4. This result was also consistent with a previous study, suggesting that GSK3β promoted HDAC4 degradation by HDAC4 phosphorylation [9]. Moreover, GSK3β was further investigated, the results of which found that GSK3β improved the morphology of NP tissues, increased proteoglycan, reduced apoptosis and pain. These results highly suggested that GSK3β inhibited the progression of IDD, which is in line with a previous study [10]. In addition, we further demonstrated that HDAC4 overexpression reversed the beneficial effects of GSK3β, which was an additional indicator of their interaction and their roles in IDD.

In addition to upstream, we also found KLF5 as a downstream mediator of HDAC4-mediated IDD. HDAC4, as a histone deacetylase, increased KLF5 expression by removing the acetylation of KLF5 [11]. KLF5 has been previously shown to promote apoptosis, a result that was consistent with our findings, suggesting that KLF5 was upregulated in IDD and may be responsible for promoting apoptosis in IDD [12]. Similarly, KLF5 was shown to promote neuropathic pain that is also consistent with our results [18]. Whether KLF5 overexpression would block the beneficial effects of GSK3β is unknown and deserved to be studied.

Furthermore, we found that KLF5 promoted the expression of ASK1, which was consistent with a previous study [12]. Moreover, we also found that ASK1 reversed the beneficial effects of GSK3β on IDD, which included decreased apoptosis and pain. These results are consistent with previous studies that illustrated the pro-apoptosis characteristics of ASK1 in NP cells and promotion of neuropathic pain [13, 14]. Our results strongly suggested that ASK1 may promote the progression of IDD.

There are a few notable limitations in this study. First, the study utilized traumatic injury model of IDD. Although this is a valid IDD model, other pathologies, such as chronic stress, can also lead to the development of IDD. Therefore, further investigations with other animal models of IDD are required for verification of our findings. Second, we used HDAC4 and GSK3β overexpression to determine their roles on IDD. We also used ASK1 overexpression to study its role on GSK3β-mediated improvement of IVD. However, the role of KLF5 remains unclear. Therefore, a more detailed study involving KLF5 overexpression and its role in IDD is encouraged.

**Fig. 7:**
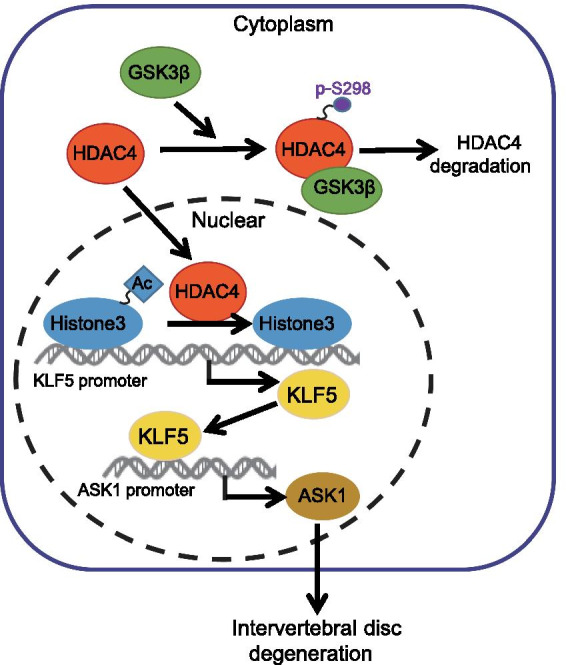
A schematic diagram showing the regulation of the GSK3β/HDAC4/KLF5/ASK1 axis in the development and progression of intravertebral disc degeneration

In conclusion, GSK3β could serve as a negative regulator of HDAC4, resulting in the alleviation of IDD, including pain (Fig. 7). This beneficial effect potentially works through the inhibition of KLF5 and ASK1. Therefore, GSK3β may be a novel treatment option for IDD.

## Materials and methods

### Ethics statement

The current study was approved by the Animal Ethics Committee in Guangxi University of Chinese Medicine, Guangxi International Zhuang Medicine Hospital. Extensive efforts were made to ensure minimal suffering of the animals used in the study.

### Establishment of a mouse IDD model

Male C57BL/6 J mice (*n* = 130, 8-week old, 20–25 g, Experimental Animal Center of Guangxi University of Chinese Medicine, Guangxi International Zhuang Medicine Hospital) were used to establish an IDD (*n* = 112) model and the remaining 18 mice served as controls. In addition, in the control group, 9 were naïve and 9 were in sham operation. Mice were anesthetized with 3% sodium pentobarbital (Cat. No. P3761, Sigma-Aldrich Chemical Company, St Louis, MO, USA). Mice were placed in a prone position, and a 20-gauge needle was used to puncture three intervertebral discs (Co6/7, Co8/9, and Co10/11) under a microscope [19]. The needle punctured through the center of the disc to the other side, rotated 180°, and held for 10 s. Co7/8 and Co9/10 served as controls. The wound was then covered with gauze. One week after puncture of the disc, mice were anesthetized with 3% sodium pentobarbital and MRI was performed to exclude severe and non-degenerated discs [20]. Mice with Pfirrmann grade 2–3 degeneration were indicative of successful model establishment (*n* = 108 out of 112). IDD mice were randomly divided into 12 groups (9 mice per group). After successful modeling, a positive control group was set. The mice in this group received treatment with the Food and Drug Administration (FDA)-approved tasquinimod (ABR-215050, CAS number: 254964-60-8) dissolved in drinking water at a daily dose of about 25 mg/kg of body weight. Lentiviral vector LV5-GFP was used for overexpression (#25999, Addgene, Cambridge, MA, USA), and pSIH1-H1-copGFP was used for gene silencing (LV601B-1, System Biosciences, Palo Alto, CA, USA). All plasmids used were made by Gene Pharma (Shanghai, China). 293 T cells were used for lentivirus packaging. 293 T cells were cultured in Roswell Park Memorial Institute (RPMI)-1640 complete medium containing 10% fetal bovine serum (FBS) and passaged every other day. A small incision was made in the left side of the mice to expose the previously punctured IDD. An adenoviral vector (1 × 10^9^ pfu/100 μL) was slowly injected into the punctured disc with a 33-gauge needle (Hamilton, Bonaduz, Switzerland). Injections were repeated 4 and 8 weeks later. NP tissues were collected for further experiments.

### NP cell culture

Mouse NP tissue samples were isolated, cut into small pieces, and treated with 0.25% streptomycin (Sigma-Aldrich Chemical Company, St Louis, MO, USA) for 30 min. After that, the tissues were treated with 0.1% type II collagenase (Invitrogen, Carlsbad, CA, USA) at 37 °C for 4 h [21]. Then, the supernatant without tissue blocks was centrifuged at 400 g for 5 min, which was resuspended in Dulbecco’s modified Eagle's medium/Ham's F-12 medium (DMEM-F12) containing 20% FBS (Gibco, Grand Island, NY, USA) at 37 °C in 5% CO_2_. The medium was changed every 48 h with DMEM-F12 medium containing 10% FBS. Cell morphology was then observed under a high-magnification inverted microscope. When cells were confluent, they underwent digestion by 0.25% trypsin/1 mM ethylenediaminetetraacetic acid (EDTA) and passaged. The third to fifth passages of NP cells were used [22]. When in logarithmic growth phase, cells were trypsinized using 1 mL of 0.25% trypsin for 3 min, and then serum-containing medium was added. After cell concentration was adjusted to 1 × 10^5^/mL, the cells were inoculated to a 6-well plate for 24 h. When cell fusion reached about 75%, cells were transfected with following plasmids: sh-negative control (NC), sh1-GSK3β, sh2-GSK3β, oe-NC, oe-GSK3β, sh1-HDAC4, sh2-HDAC4, using Lipofectamine 2000 reagents (Invitrogen Inc., Carlsbad, CA, USA).

### mRNA expression determined by RT-qPCR

TRIzol reagents (Shanghai Haling Biotechnology Co., Ltd., Shanghai, China) were used to extract the total RNA from cells in each group. The RNA concentration, purity, and integrity were determined by Nano-Drop ND-1000 spectrophotometry and 1% agarose gel electrophoresis. Reverse transcription reaction was performed on a PCR amplification equipment to synthesize a complementary DNA (cDNA) template using a reverse transcription kit (Transgene Biotech, Beijing, China). Reverse transcription system (20 μL) was performed according to the instructions of EasyScript First-Strand cDNA Synthesis SuperMix (#AE301-02, Transgene Biotech). Reaction solution was collected for real-time quantitative PCR by SYBR® Premix Ex TaqTM II Kit (TaKaRa, Dalian, China) using ABI 7500 system. Primer sequences are shown in Table 1. Relative mRNA expression of target genes was calculated by the 2^−ΔΔCt^ method. Each sample was tested in triplicate.

**Table 1:** Primer sequences for RT-qPCR

Primer	Upstream sequence (5′–3′)	Downstream sequence (5′–3′)
GSK3β	CCCAAGCTTATGGAATATTATCTTGT	CGGGATCCGTTGAGGTAACCTCTGC
HDAC4	GCAGAGGTTGAATGTGAGCA	GGAAGAAGTTCCCATCGTCA
ASK1	CAAGCGTGAGACTCGTGATCCTTC	AGGCTGCTGCACCGGCTTGCAGCT
KLF5	CACTACTGCGATTACCCTG	GGTCTGTCATTTGAGGGAG
GAPDH	ATCACTGCCACCCAGAAGAC	ATCCACGACGGACACATTGG

### Protein expression determined by Western blotting

Total tissue protein was extracted by a radioimmunoprecipitation assay (RIPA) kit (R0010, Beijing Solarbio Science & Technology Co., Ltd., Beijing, China). Protein concentration was determined using a bicinchoninic acid (BCA) protein assay kit (GBCBIO Technologies, Guangzhou, China). Protein (40 µg) from each sample was loaded and separated with 10% sodium dodecyl sulfate-polyacrylamide gel electrophoresis (SDS-PAGE). Proteins were transferred to a polyvinylidene fluoride (PVDF) membrane and blocked with Tris-buffered saline Tween-20 (TBST) solution containing 5% bovine serum albumin (BSA) at room temperature. Membranes underwent incubation with primary antibodies against GSK3β (1:1000, ab2602, Abcam Inc., Cambridge, UK), phosphorylated (p)-HDAC4, ASK1 (1:1000, ab45178, Abcam Inc., Cambridge, UK), HDAC4 (1:400, ab79521, Abcam Inc., Cambridge, UK), KLF5 (1:500, ab137676, Abcam Inc., Cambridge, UK), cleaved caspase-3 (1:1000, ab2302, Abcam Inc., Cambridge, UK), Bcl-2 (1:2000, ab182858, Abcam Inc., Cambridge, UK), Bax (1:2000, ab182733, Abcam Inc., Cambridge, UK), and glyceraldehyde-3-phosphate dehydrogenase (GAPDH) (1:2500, ab9485, Abcam Inc., Cambridge, UK) at 4 °C overnight. Membranes were subsequently incubated with secondary antibody goat anti-rabbit immunoglobulin G (IgG) antibody (1:5000, ab205718, Abcam Inc., Cambridge, UK) or goat anti-mouse IgG antibody (1:5000, ab205719, Abcam Inc., Cambridge, UK) and at room temperature. Protein bands were developed using enhanced chemiluminescence reagents. Gray intensity in each protein band was analyzed by ImageJ software and normalized to internal reference protein GAPDH. Each sample was tested in triplicate.

### ChIP assay

ChIP detection was performed using an EZ-Magna ChIP kit (EMD Millipore, Burlington, MA, USA). In brief, cells were fixed with 4% paraformaldehyde and incubated with glycine for 10 min to produce DNA–protein crosslinks. Then, the cells were lysed using cell lysis buffer and nuclear lysis buffer, which were subsequently sonicated to produce 200–300 bp chromatin fragments. Next, the cell lysates were immunoprecipitated with magnetic protein A beads bound to the antibodies: HDAC4 (1:400, ab79521, Abcam Inc., Cambridge, UK), acetyl-histone H3 (Lys9, SAB5600232, Sigma-Aldrich Chemical Company, St Louis, MO, USA), KLF5 (1:500, ab137676, Abcam Inc., Cambridge, UK), and ASK1 (1:300, ab45178, Abcam Inc., Cambridge, UK). Meanwhile, Rabbit IgG (ab171870, Abcam Inc., Cambridge, UK) was used as a negative control. Finally, the KLF5 and ASK1 promoters in the precipitated DNA were analyzed and quantified by RT-qPCR.

### Co-IP assay

Transfected cells were treated in a lysis buffer (50 mM Tris–HCl, pH 7.4; 150 mM NaCl; 10% glycerol; 1 mM EDTA; 0.5% NP-40; a protease inhibitor mix), and cell debris was removed by centrifugation. The clear cell lysate was incubated with 1 μG HDAC4 antibody (1:500, ab79521, Abcam Inc., Cambridge, UK) or anti-GSK3β (1:500, ab2602, Abcam Inc., Cambridge, UK) and 15 μL protein A/G beads (Santa Cruz Biotechnology, Santa Cruz, CA, USA) for 2 h. After washing, beads were boiled at 100 °C for 5 min. Proteins were separated by SDS-PAGE, transferred to a nitrocellulose membrane (Millipore, Temecula, CA, USA), and then immunoblotted. To detect endogenous protein interactions, cells were lysed in ice-cold lysis buffer. The cleared cell lysate underwent incubation with 5 µg of anti-HDAC4 antibody and 20 μL of protein A/G beads at 4 °C overnight. Anti-HDAC4 antibody or anti-GSK3β antibody was used to detect endogenous levels of HDAC4 or GSK3β, respectively.

### IP assay

NP cells with HADC4 overexpression were transfected with the plasmid expressing Flag-KLF5 and cultured for 48 h, and the IP was purified by Flag antibody. The precipitate was washed with pre-lysis buffer and analyzed by Western blotting. Antibodies used in immunoblotting and co-immunoprecipitation were HADC4 (1:500, ab79521, Abcam Inc., Cambridge, UK), anti-acetyl-histone H3 (Lys9, SAB5600232, Sigma-Aldrich Chemical Company, St Louis, MO, USA).

### Apoptosis determined by terminal deoxynucleotidyl transferase-mediated dUTP nick end labeling (TUNEL) assay

TUNEL staining was used to detect apoptotic DNA fragments. Degenerative disk NP cells were cultured with 3% H_2_O_2_ and 0.1% Triton X-100 for 10 min and washed 3 times with phosphate-buffered saline (PBS). Cells were then fixed with 4% paraformaldehyde in situ following the instructions in an apoptosis detection kit (Roche, Basel, Switzerland) and stained with 4′,6-diamidino-2-phenylindole (DAPI). Apoptosis was observed under a light microscope (Olympus BX61, Tokyo, Japan). The experiment was repeated three times.

### H&E staining

Mouse intervertebral disc tissues were fixed with 4% paraformaldehyde, embedded in paraffin, and cut into 5-μm sections. Tissue sections were then stained with Alcian blue (BHBT, Shanghai, China) and Safranin O fast green (Beijing Solarbio Science & Technology Co., Ltd., Beijing, China). In H&E staining, tissue sections were dewaxed with xylene I for 10 min and xylene II for 5 min, followed by rehydration with anhydrous alcohol for 1 min, 95% alcohol for 1 min, and 85% alcohol for 1 min. After that, tissue sections were stained with hematoxylin (Beyotime Biotechnology Co. Ltd., Shanghai, China) for 5 min and then with eosin (Beyotime Biotechnology Co. Ltd., Shanghai, China) for 2 min. Tissues were then dehydrated with 85% alcohol dehydration for 20 s, 95% alcohol for 1 min, absolute alcohol I for 2 min, anhydrous alcohol II for 2 min, xylene I for 2 min, and xylene II for 2 min, and sealed with neutral or Canadian balsam resin. Finally, the sections were observed under a light microscope (DMI3000, Leica, Wetzlar, Germany).

### Safranin O fast green staining

Mouse intervertebral disc tissue sections were dewaxed and hydrated as above and stained for 3 min with hematoxylin. Sections were differentiated with 1% hydrochloric acid alcohol for 5 s and stained with 0.02% fast green solution for 10 min [23]. After washing with 1% acetic acid, tissues were stained with Safranin O aqueous solution for 3 min, dehydrated, cleared with xylene, and sealed with neutral resin. Subsequently, the tissue sections were observed under a microscope.

### Intervertebral DHI calculation

Mice were anesthetized, and their limbs and tails were secured so that their tail muscles were relaxed vertically. The Faxitron Cabinet X-ray system (Faxitron Corp, Wheeling, IL) was used to scan the mice on week 4 and 8 after modeling. Disc height was obtained and DHI was calculated [24]. The change of DHI value is expressed as %DHI, which is the ratio between postoperative and preoperative DHI.

### Assessment of pain

Mice were habituated to the test environment for at least 2 days. Mechanical sensitivity was tested by the von Frey Hair (Woodland Hills, Los Angeles, CA, USA) test. Prior to testing, mice were placed in a metal mesh box for 30 min. A series of von Frey hairs with logarithmically increasing stiffness were used to vertically stimulate the plantar surface of each hind paw, and the paw withdrawal observed. Each mouse was tested 3 times, and the average of the thresholds was calculated.

To demonstrate thermal hyperalgesia, withdrawal latency of mouse feet to thermal stimulation was measured. An analgesic device (Ugo Basile, Italy) was used to provide a heat source, and animals were placed in boxes with smooth and temperature-controlled glass floors. The heat was transmitted through the heat source to the hind paw. When the hind paw was retracted, the stimulus was turned off (or removed after 20 s to prevent tissue damage). In all experiments, the intensity of the thermal stimulus remained constant. In control animals, withdrawal latency was 9–14 s. Thermal stimuli were delivered to each hind paw three times at 5–6-min intervals [14].

### Statistical analysis

Data analysis was performed by SPSS 21.0 statistical software (IBM Corp. Armonk, NY, USA). Data are expressed as mean ± standard deviation (SD). Data from two groups were compared by unpaired *t *test. Data from multiple groups were compared by one-way analysis of variance (ANOVA) and post hoc Tukey’s test. Data comparison between groups at different time points was made using repeated-measures ANOVA and post hoc Bonferroni test. Differences were considered significant when *p* < 0.05.

## Supplementary Information

**Additional file 1.** Figure S1 H&E staining of disc tissues in each group (200 ×). A: HDAC4 promotes IDD by elevating ASK1 expression. B: GSK3β alleviates IDD by inhibiting HDAC4 expression. C: GSK3β alleviates IDD by inhibiting ASK1 expression.

**Additional file 2.** Figure S2 Safranin O fast green staining of disc tissues in each group (200 ×). A: HDAC4 promotes IDD by elevating ASK1 expression. B: GSK3β alleviates IDD by inhibiting HDAC4 expression. C: GSK3β alleviates IDD by inhibiting ASK1 expression.

## Data Availability

The datasets generated and/or analyzed during the current study are available from the corresponding author on reasonable request.

## Abbreviations

IDD: Intervertebral disc degeneration
DHI: Disc height index
HDAC4: Histone deacetylase 4
KLF5: Kruppel-like factor 5
NP: Nucleus pulposus
GSK3β: Glycogen synthase kinase-3β
ASK1: Apoptosis signal-regulating kinase 1
ChIP: Chromatin immunoprecipitation
Co-IP: Co-immunoprecipitation
HE: Hematoxylin and eosin
RPMI: Roswell Park Memorial Institute
oe: Overexpression
NC: Negative control
sh: Short hairpin
EDTA: Ethylenediaminetetraacetic acid
BSA: Bovine serum albumin
TUNEL: Terminal deoxynucleotidyl transferase-mediated dUTP-biotin nick end labeling
PBS: Phosphate-buffered saline
DMEM-F12: Dulbecco’s modified Eagle’s medium/Ham’s F-12 medium
FBS: Fetal bovine serum
cDNA: Complementary DNA
RT-qPCR: Reverse transcription quantitative polymerase chain reaction
GAPDH: Glyceraldehyde-3-phosphate dehydrogenase
RIPA: Radioimmunoprecipitation assay
BCA: Bicinchoninic acid
PVDF: Polyvinylidene fluoride
SDA-PAGE: Sodium dodecyl sulfate-polyacrylamide gel electrophoresis
TBST: Tris-buffered saline with Tween-20
DAPI: 4′,6-Diamidino-2-phenylindole
Bax: Bcl-2-associated X protein
Bcl-2: B-cell lymphoma 2
IgG: Immunoglobulin G
SD: Standard deviation
ANOVA: Analysis of variance

## References

[1] Vergroesen PP, Kingma I, Emanuel KS, Hoogendoorn RJ, Welting TJ, van Royen BJ (2015). Mechanics and biology in intervertebral disc degeneration: a vicious circle. Osteoarthr Cartil.

[2] Rider SM, Mizuno S, Kang JD (2019). Molecular mechanisms of intervertebral disc degeneration. Spine Surg Relat Res.

[3] Andersson GB (1999). Epidemiological features of chronic low-back pain. Lancet.

[4] Frank A (1993). Low back pain. BMJ.

[5] Kadow T, Sowa G, Vo N, Kang JD (2015). Molecular basis of intervertebral disc degeneration and herniations: what are the important translational questions?. Clin Orthop Relat Res.

[6] Oikarinen J (1991). Histone H1 and the regulation of transcription by nuclear receptors. FEBS Lett.

[7] Mizzen CA, Allis CD (1998). Linking histone acetylation to transcriptional regulation. Cell Mol Life Sci.

[8] Zheng H, Hu S, Cao J, Yao L, Zhang N (2019). Long non-coding RNA TUG1 alleviates LPS-induced injury of PC-12 cells by down-regulating microRNA-127. Exp Mol Pathol.

[9] Cernotta N, Clocchiatti A, Florean C, Brancolini C (2011). Ubiquitin-dependent degradation of HDAC4, a new regulator of random cell motility. Mol Biol Cell.

[10] Wang X, Wang B, Zou M, Li J, Lu G, Zhang Q (2018). CircSEMA4B targets miR-431 modulating IL-1beta-induced degradative changes in nucleus pulposus cells in intervertebral disc degeneration via Wnt pathway. Biochim Biophys Acta Mol Basis Dis.

[11] Wang Z, Sun L, Jia K, Wang H, Wang X (2019). miR-9-5p modulates the progression of Parkinson’s disease by targeting SIRT1. Neurosci Lett.

[12] Tarapore RS, Yang Y, Katz JP (2013). Restoring KLF5 in esophageal squamous cell cancer cells activates the JNK pathway leading to apoptosis and reduced cell survival. Neoplasia.

[13] Liu Y, Guan H, Zhang JL, Zheng Z, Wang HT, Tao K (2018). Acute downregulation of miR-199a attenuates sepsis-induced acute lung injury by targeting SIRT1. Am J Physiol Cell Physiol.

[14] Zhou D, Zhang S, Hu L, Gu YF, Cai Y, Wu D (2019). Inhibition of apoptosis signal-regulating kinase by paeoniflorin attenuates neuroinflammation and ameliorates neuropathic pain. J Neuroinflammation.

[15] Zheng Q, Li XX, Xiao L, Shao S, Jiang H, Zhang XL (2019). MicroRNA-365 functions as a mechanosensitive microRNA to inhibit end plate chondrocyte degeneration by targeting histone deacetylase 4. Bone.

[16] Wang Y, Xia Y, Hu K, Zeng M, Zhi C, Lai M (2019). MKK7 transcription positively or negatively regulated by SP1 and KLF5 depends on HDAC4 activity in glioma. Int J Cancer.

[17] Liu Y, Lin J, Wu X, Guo X, Sun H, Yu B (2019). Aspirin-mediated attenuation of intervertebral disc degeneration by ameliorating reactive oxygen species in vivo and in vitro. Oxid Med Cell Longev.

[18] Jiang BC, Zhang WW, Yang T, Guo CY, Cao DL, Zhang ZJ (2018). Demethylation of G-protein-coupled receptor 151 promoter facilitates the binding of kruppel-like factor 5 and enhances neuropathic pain after nerve injury in mice. J Neurosci.

[19] Piazza M, Peck SH, Gullbrand SE, Bendigo JR, Arginteanu T, Zhang Y (2018). Quantitative MRI correlates with histological grade in a percutaneous needle injury mouse model of disc degeneration. J Orthop Res.

[20] Pfirrmann CW, Metzdorf A, Zanetti M, Hodler J, Boos N (2001). Magnetic resonance classification of lumbar intervertebral disc degeneration. Spine (Phila Pa 1976).

[21] Wu Q, Mathers C, Wang EW, Sheng S, Wenkert D, Huang JH (2019). TGF-beta Initiates beta-catenin-mediated CTGF secretory pathway in old bovine nucleus pulposus cells: a potential mechanism for intervertebral disc degeneration. JBMR Plus.

[22] Song Y, Dou H, Li X, Zhao X, Li Y, Liu D (2017). Exosomal miR-146a contributes to the enhanced therapeutic efficacy of interleukin-1beta-primed mesenchymal stem cells against sepsis. Stem Cells.

[23] Leung VY, Chan WC, Hung SC, Cheung KM, Chan D (2009). Matrix remodeling during intervertebral disc growth and degeneration detected by multichromatic FAST staining. J Histochem Cytochem.

[24] Liao Z, Luo R, Li G, Song Y, Zhan S, Zhao K (2019). Exosomes from mesenchymal stem cells modulate endoplasmic reticulum stress to protect against nucleus pulposus cell death and ameliorate intervertebral disc degeneration in vivo. Theranostics.

